# Acquired Immunodeficiency Syndrome Presented as Atypical Ocular Toxoplasmosis

**DOI:** 10.1155/2021/5512408

**Published:** 2021-05-01

**Authors:** Elias Khalili Pour, Hamid Riazi-Esfahani, Nazanin Ebrahimiadib, Violet Zaker Esteghamati, Mohammad Zarei

**Affiliations:** ^1^Eye Research Center, Farabi Eye Hospital, Tehran University of Medical Sciences, Tehran, Iran; ^2^Imam Khomeini General Hospital, School of Medicine, Tehran University of Medical Sciences, Tehran, Iran

## Abstract

**Background:**

The aim is to report an atypical presentation of ocular toxoplasmosis which led to the diagnosis of Acquired Immunodeficiency Syndrome (AIDS). *Case Report*. The 38-year-old woman was referred with metamorphopsia and reduced vision in the right eye over the past 3 weeks. Slit-lamp examination revealed granulomatous keratic precipitates (KPs), 2+ cells in the anterior chamber, and posterior synechiae. Fundus examination was remarkable for a white patch surrounding a scar, inferonasal to the optic disc with fibrous bands emanating from the lesion, and the retina around this region was detached with considerable extension towards the periphery, while no breaks could be appreciated. She mentioned anorexia and losing 10 kg in the past three months, and signs of anemia like paleness of face skin, bed nails, and bilateral angular cheilitis were observed on systemic evaluation. The results of the patient's complete blood count revealed anemia and leukopenia and CD4 lymphocytes: 79 cells/*μ*L. Enzyme-linked immunosorbent assays (ELISA) for HIV antibodies came back positive which was later confirmed with the Western blot test. Brain magnetic resonance imaging (MRI) showed multiple ring-enhancing lesions in both cerebral cortices. The patient underwent antitoxoplasmosis and anti-HIV treatment and serous retinal detachment completely resolved.

**Conclusion:**

This report highlights the fact that sometimes, the eyes are the site of the first presentation of a systemic life-threatening condition and emphasizes the role of ophthalmologists in such cases. In cases of atypical presentation, appropriate laboratory tests and CNS imaging should be requested. Systemic treatment with antitoxoplasmosis regimens and highly active antiretroviral therapy (HAART) is mandatory in AIDS patients with ocular toxoplasmosis.

## 1. Background

Since the 1950s, ocular toxoplasmosis has been reported as the most common cause of infectious uveitis worldwide. *Toxoplasma gondii* is a ubiquitous obligate intracellular microorganism, which is estimated to affect one-third of the world's population [1–3].

Active ocular toxoplasmosis is characterized by a necrotizing retinochoroiditis lesions accompanied by localized or diffuse vitritis. In immunocompetent patients, lesions are usually self-limited and resolve within two months. Atypical forms of ocular toxoplasmosis such as diffuse outer retinitis, which can mimic acute retinal necrosis (ARN), punctuate outer retinal toxoplasmosis, occlusive retinal vasculitis, neuroretinitis, scleritis, and exudative retinal detachment, have been reported [4]. Atypical lesions may occur bilaterally and have been reported in the immunocompromised, neonates, and elderly patients [5].

This parasite can be fatal in immunocompromised individuals, such as HIV/AIDS patients especially those with CD4 T lymphocyte cell counts less than 200 cells/*μ*L. In such a setting, reactivation of opportunistic organisms like *Toxoplasma gondii* can occur with multiorgan involvement, especially central nervous system (CNS), and with much lower incidence, ocular involvement [5–7].

The central nervous system, as the most common site of Toxoplasma reactivation, can be involved in about 30% of patients with AIDS, especially in those who are treatment-naive and Toxoplasma seropositive with CD4 < 100 cells/*μ*L [8–11].

Here, we report an atypical presentation of ocular toxoplasmosis with a serous retinal detachment which led to the diagnosis of AIDS. The serous retinal detachment resolved with antitoxoplasmosis treatments and HAART.

## 2. Case Report

**Figure 1:**
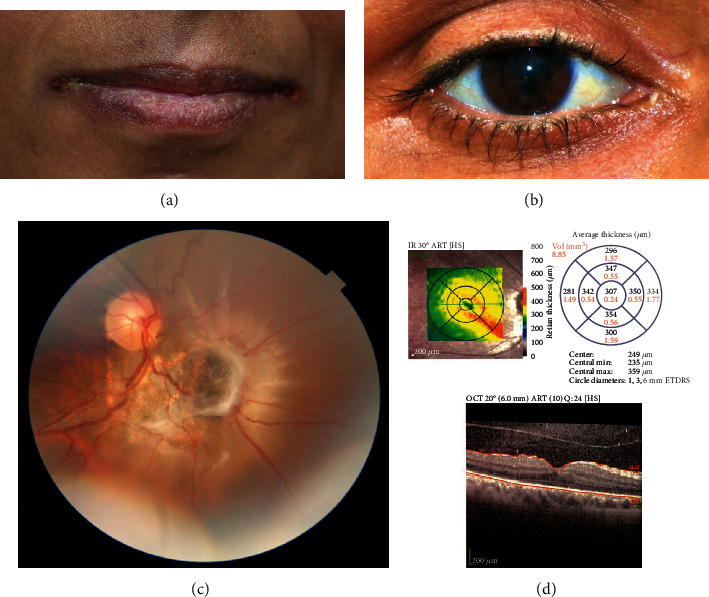
Clinical features of the patient at the time of presentation. Bilateral angular cheilitis (a), posterior synechiae, and keyhole-shaped pupil (b), In the right eye inferonasal to the optic disc, an annular white patch centered with a pigmented scar is seen. White subretinal bands are extending from the lesion. The size of the lesion is about three-disc diameter, and the adjacent retina is detached (c). Macular optical coherence tomography of the right eye demonstrates evidence of vitritis, detached posterior hyaloid, and a fine epiretinal membrane nasal to the fovea (d).

The 38-year-old woman was referred to the retina department of Farabi Eye Hospital with metamorphopsia and reduced vision in the right eye over the past 3 weeks. At the time of the presentation, she mentioned anorexia and losing 10 kg in the past three months, and signs of anemia like paleness of face skin, bed nails, and bilateral angular cheilitis were observed (Figure 1(a)). Uncorrected visual acuity in the right eye was counting fingers at two meters. Posterior synechia (PS) and pigment deposit over the anterior crystalline lens capsule precluded precise refraction in this eye. Slit-lamp examination revealed granulomatous keratic precipitates (KPs) distributed in Arlt's triangle, 2+ cells in the anterior chamber, and a relatively broad-based PS causing a keyhole appearance in the pupil (Figure 1(b)). The crystalline lens was clear and 2+ cells were present in the anterior vitreous. Fundus examination was remarkable for a white patch surrounding a scar, inferonasal to the optic disc with three-disc diameter size. Some fibrous bands were emanating from the lesion, and the retina around this region was detached with considerable extension towards the superior, nasal, and inferior periphery, while no breaks could be appreciated (Figure 1(c)). The vision in the left eye was 20/20, and ophthalmic examination was unremarkable in this eye.

Spectral-domain optical coherence tomography (SD-OCT) (Spectralis Heidelburg Germany) disclosed evidence of vitritis, a detached posterior hyaloid face, and a fine epiretinal membrane (ERM) nasal to the fovea in the right eye (Figure 1(d)).

She denied using any medications or having other illnesses. Considering systemic symptoms and signs and due to involuntary weight loss, the patient underwent a comprehensive infectious, neoplastic, and rheumatologic workup. The results of the patient's complete blood count (CBC) are summarized in Table 1. Based on the ocular findings, additional tests were ordered for *Toxoplasma gondii* antibodies which revealed the presence of IgG antibodies (18 IU/mL; reference range: positive ≥ 12 IU/mL, suspicious 10-11 9 IU/mL, negative ≤ 9 IU/mL) while IgMs were absent (0.1 IU/mL; reference range: positive ≥ 0.65 IU/mL, suspicious 0.55-0.64 IU/mL, negative ≤ 0.55 IU/mL).

Imaging studies including chest-X-ray, abdominal sonography, and age-related neoplastic workup including mammography, Pap smear, and colonoscopy were unremarkable except for nonspecific polyps in the patient's large intestine.

Enzyme-linked immunosorbent assays (ELISA) for HIV antibodies came back positive which was later confirmed with the Western blot test.

**Figure 2:**
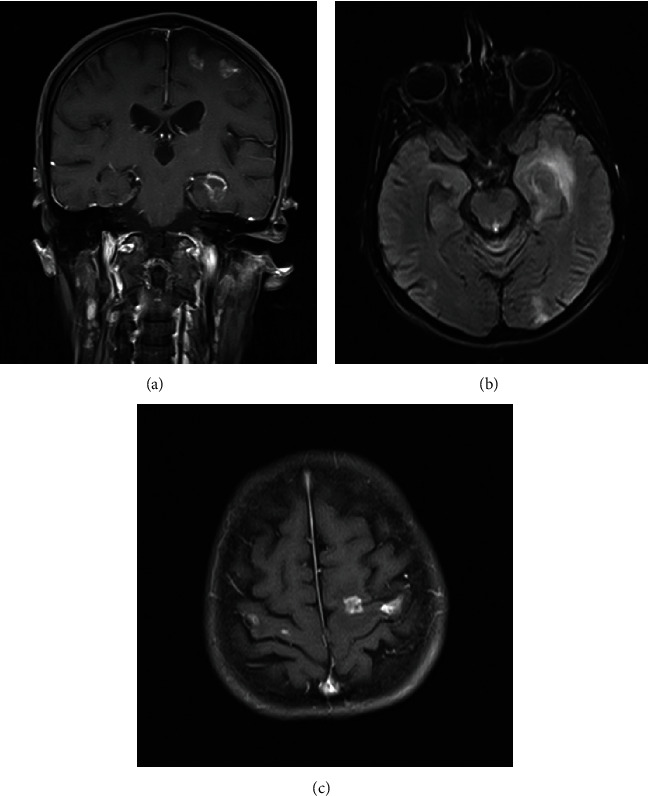
Magnetic resonance imaging (MRI): T1 with contrast in coronal (a) and axial planes (b, c): multiple ring-enhancing lesions in the left and right cerebral cortices.

Once the diagnosis of toxoplasmic retinochoroiditis in an immunosuppressed patient was established, treatment with trimethoprim/sulfamethoxazole (TMP/SMX) 160/800 mg twice daily was commenced. Brain magnetic resonance imaging (MRI) showed multiple ring-enhancing lesions in both cerebral cortices (Figure 2). Meanwhile, three weeks after initial ophthalmic presentations, the patient developed a right hemiparesis. Due to the numerous lesions in the patient's motor cortex in the left and right lobes, the diagnosis of toxoplasmic encephalitis was made for the patient.

**Figure 3:**
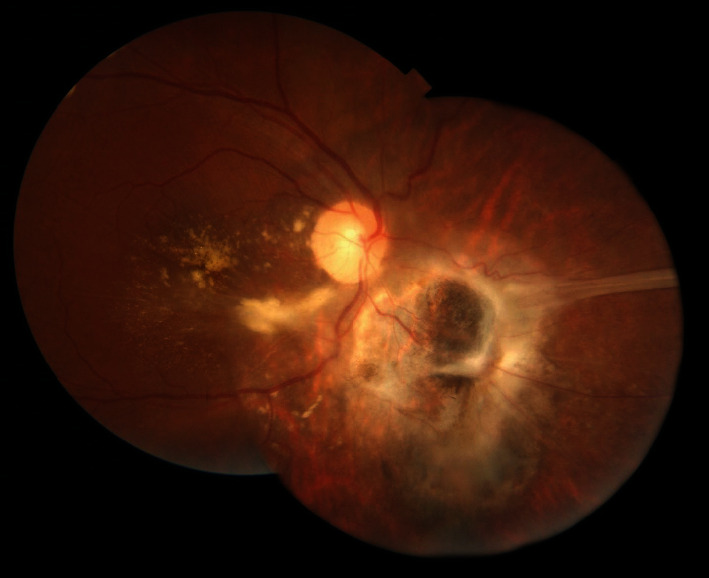
Montage fundus photography of the patient's right eye three months after initiation of antitoxoplasmosis treatment and HAART shows complete retinal reattachment. A retinal fold extends from the lesion to the nasal periphery. Subretinal exudates are seen in the inferior area of the disc and around the macula.

Highly active antiretroviral therapy (HAART) was added to her antitoxoplasmosis treatment four weeks after initial presentation and the dose of trimethoprim/sulfamethoxazole (TMP/SMX) increased to 160/800 mg/three times a day and azithromycin 250 mg/daily was added to the antitoxoplasmic regimen. The systemic steroid was not added to the patient's treatment regimen due to concerns about the patient's immunosuppression. Two months after initial presentation, vitreous inflammation decreased substantially, subretinal fluid gradually resolved, and the uncorrected visual acuity improved to 20/100 (Figure 3). Yellow subretinal hard exudates appeared in the perifoveal area with complete reattachment of the retina in three months. Two months after starting HAART, the patient's hemiparesis improved significantly during this time.

## 3. Discussion

The presence of cells in the anterior chamber and vitreous cavity, large Kps, PS, and a patch of retinitis adjacent to a pigmented retinochoroidal scar are clinical clues to the diagnosis of toxoplasmic retinochoroiditis. According to the fundus examination, weight loss, anemia, and leukopenia of the patient, internal medicine and infectious disease service consultation were requested. The patient's neuroimaging and the appearance of typical ring-enhanced lesions besides positive HIV test and diminished CD4+ cells suggested a possible diagnosis of toxoplasmosis in the patient, and therefore, no intraocular fluid PCR test requested for the patient. Positive Toxoplasma IgG antibody along with negative IgM indicates infection with the organism at some time [2, 12, 13]. According to a meta-analysis conducted by Borna et al., for estimating the level of immunity to toxoplasmosis in reproductive ages in Iran, the overall estimation for the prevalence of antitoxoplasma total antibody was 39.9% (95% CI: 26.1-53.7) [14].

However, this case shows some ocular and systemic features that are not commonly expected in the typical case of toxoplasmic retinochoroiditis. In this case, pursuing these atypical features led us to the diagnosis of AIDS.

The first red flag was the presence of an extensive retinal detachment with tractional bands surrounding the central scar and no responsible break which could be suggestive of tractional and/or exudative retinal detachment in our case. Although tractional, exudative, and rhegmatogenous retinal detachments can occur in ocular toxoplasmosis, such reports are limited; the reported prevalence of retinal detachment in acquired toxoplasmosis varies between 2.5 and 11 percent which is mostly of tractional or rhegmatogenous type [13, 15–17].

There are several reports on the management of retinal detachment in the setting of ocular toxoplasmosis. Among the 193 patients with ocular toxoplasmosis, Kianersi et al. reported five patients with RD: three rhegmatogenous and two tractional. All five patients underwent vitrectomy or scleral buckling surgery. It is well known that severe intraocular inflammation can lead to tractional retinal detachment. With the progression of traction, a retinal hole and subsequent rhegmatogenous detachment may develop. Another possible mechanism of hole formation is the occurrence of full-thickness necrosis in the retinitis patch [17].

In another study, Al-Zahrani et al. reported a 24-year-old man with exudative RD secondary to reactivation of a toxoplasmosis lesion, which was successfully managed with antitoxoplasmosis treatment [15].

In a study by Faridi et al., on thirty-five eyes of 28 patients with ocular toxoplasmosis and sufficient follow-up, they identified 4 eyes (11 percent) with secondary RD, which was either rhegmatogenous, tractional, or a combination of the two. All eyes with retinal detachment underwent surgical repair of which 50% developed recurrent RDs (two of 4 patients). They concluded that handling ocular toxoplasmosis and related RDs may necessitate several measures, including surgical management, systemic therapy, and intravitreal injections. Formation of new breaks or PVR-related complications may necessitate multiple surgeries in cases with recurrent RD [16]. Our case represents the exudative detachment with no distinct retinal break. Observation of a retinal fold may advocate the presence of a tractional component in this case. The resolution of RD following an antitoxoplasmic regimen and HAART and without surgical intervention may suggest that decision upon surgery should not be rushed in such patients, and a period of close follow-up visits for ocular examination combined with appropriate systemic treatment may be warranted.

Angular cheilitis and significant weight loss are not expected to occur in typical ocular toxoplasmosis. The presence of angular cheilitis calls for investigating the possibility of anemia and its underlying etiology. In our case, CBC revealed hypochromic microcytic anemia suggestive of iron-deficiency anemia and leukopenia which necessitates a thorough investigation for immunodeficiency.

Increased energy expenditure happens in the setting of opportunistic infections. Weight loss in patients with HIV is associated with poor prognosis, increased risk of disease progression, and opportunistic pathology [18].

In a study by Neves et al. on 37 immunocompetent patients with acute acquired toxoplasmosis, the frequency of systemic manifestations was inspected. Weight loss, anemia, and leukopenia were seen in 62.2%, 10.8%, and 16.2% of patients, respectively [12]. Although our patient was not a case of acquired toxoplasmosis and the results of the study by Neves et al. could not be generalized to reactivated congenital toxoplasmosis, another similar study could investigate the possibility of anemia, leukopenia, and weight loss in this group of patients.

The possibility of toxoplasmic encephalitis should be investigated in every patient with HIV and retinochoroidal toxoplasmosis [19, 20]. The study of choice for this purpose is brain MRI with contrast that can illustrate characteristic enhancing ring lesions. While not pathognomonic, the presence of several brain abscesses is the most characteristic feature of *T. gondii* infection in AIDS patients. Autopsy usually shows a global involvement of both hemispheres, though the basal ganglia and the corticomedullary junction are the most common sites of involvement [9–11].

This report highlights the fact that sometimes the eyes are the site of the first presentation of a systemic life-threatening condition and emphasizes the role of ophthalmologists in such cases. Judicious ocular and systemic evaluation of patients with ocular toxoplasmosis are of utmost importance. In cases of atypical presentation, appropriate laboratory tests and CNS imaging should be requested. Systemic treatment with antitoxoplasmosis regimens and HAART is mandatory in AIDS patients with ocular toxoplasmosis. Treatment options for retinal detachment in this setting should be meticulously approached.

## Figures and Tables

**Table 1:** The results of the patient's CBC-diff.

Blood component		Reference range
White blood cells	3400	4500-11,000/mm^3^
Red blood cells	4.19	Male: 4.3-5.9 million/mm^3^ Female: 3.5-5.5 million/mm^3^
Hemoglobin	8.1	Male: 13.5-17.5 g/dL Female: 12.0-16.0 g/dL
Hematocrit	26.8	Male: 41%-53% Female: 36%-46%
Mean corpuscular volume	64.0	80-100 *μ*m^3^
Mean corpuscular hemoglobin	19.3	25.4-34.6 pg/cell
Mean corpuscular hemoglobin concentration	30.2	31%-36% Hb/cell
Platelets	236000	150,000-400,000/mm^3^
Neutrophil	61.7	40-60%
Lymphocytes	27.9	20-40%
Monocytes	7.8	4-8%
Eosinophil	2.6	1-3%
Basophil	0	0-1%
CD4 lymphocytes	79 cells/*μ*L	500-1400 cells/*μ*L

## Data Availability

The data generated during the present study is available upon request from the corresponding author.
